# Evaluation of the PrimeFlow RNA assay as a method of detection of SARS-CoV-2 single and dual Infections

**DOI:** 10.1007/s10616-023-00608-9

**Published:** 2023-11-24

**Authors:** Shollie M. Falkenberg, Alexa Buckley, Paola Boggiatto

**Affiliations:** 1 Ruminant Disease and Immunology Research Unit, National Animal Disease Center, USDA, Agricultural Research Service, Ames, IA USA; 2 College of Veterinary Medicine, Department of Pathobiology, Auburn University, Auburn, AL USA; 3 Virus and Prion Research Unit, National Animal Disease Center, USDA, Agricultural Research Service, Ames, IA USA; 4 Infectious Bacterial Diseases of Livestock Research Unit, National Animal Disease Center, USDA, Agricultural Research Service, Ames, IA USA

**Keywords:** SARS-CoV-2, PrimeFlow RNA assay, Cell culture, Variants

## Abstract

Given the implications of increased transmissibility, virulence, host range, and immune escapes of emerging variants of SARS-CoV-2, developing in vitro models that allow for detection of variants and differences in infection dynamics is important. The objective of this study, was to evaluate the PrimeFlow RNA in-situ assay as a method of detection for multiple strains of SARS-CoV-2. Evaluation of detection and infection statuses included single infections with an Alpha, Delta, or Omicron variants and dual infections with Alpha/Omicron or Delta/Omicron. RNA probes specific for the Spike protein coding region, were designed (omicron or non-omicron specific). SARS-CoV-2 RNA was detected in greater frequency in the Vero E6 and minimally in the fetal deer testicle cell lines by flow cytometry using this approach for viral detection of multiple variants. Most evident in the Vero E6 cells, 24 h post infection both Alpha and Delta predominated over Omicron in dual infections. This is the first report using the PrimeFlow assay for the detection of SARS-CoV-2 at the single-cell level and as a potential model for competition of variants utilizing infection dynamics in cell culture.

## Introduction

 Severe acute respiratory syndrome coronavirus 2 (SARS-CoV-2) is a positive sense, single-stranded RNA virus within the *Betacoronavirus* genus, of the family *Coronaviridae* (Kim et al. 2020). Since its emergence in 2019, SARS-CoV-2 has continued to evolve and generate new variants. In vitro models provide an opportunity to evaluate replication or infection dynamics of various SARS-CoV-2 variants to determine viral strains that may predominate. This is especially important given that a single amino acid change in the S protein at position 614 (S(D614G) was identified in a small fraction of sequenced samples and within a few weeks became the predominant variant worldwide. This fitness advantage conferred by this single amino acid change was supported by increases in infectivity and viral load in vitro and in vivo (Hou et al. 2020; Plante et al. 2021; Zhou et al. 2021).

Therefore, the emergence and significance of variants have highlighted the need to better understand the role of fitness in predominance of variants or co-infection. Recently, genomic surveillance has described co-infections in high virus co-circulation periods (Combes et al. 2022). While co-infections with SARS-CoV-2 have been described, few methods to evaluate co-infection dynamics and viral fitness have been reported. Traditional methods that can be used to characterize or evaluate virus competition and fitness include bioinformatic approaches, DNA sequencing, polymerase chain reaction (PCR), and immunodetection methods. A drawback of these methods is that they provide a consensus of the overall population or presence and absence but lack the ability to define viral dynamics at the single cell level. Collectively, this highlights the need to develop methods/models to better assess infection dynamics beyond the presence, absence, and consensus and provide quantification for SARS-CoV-2.

The goal of this research was to evaluate the PrimeFlow assay as method to detect infection with different SARS-CoV-2 variants following in vitro infection of two cell lines Vero E6 and fetal deer testicle cell lines that differ in infection dynamics and rates to determine the frequency of viral RNA detection for each variant. This method would allow for quantitative assessment through frequency of infected cells via detection of viral RNA of single and co-infected cells using Alpha, Omicron and Delta variants.

## Materials and methods

### Viruses

The USA-WA1/2020 (BEI NR-58,221 Alpha variant), hCoV-19/USA/MD-HP20874/2021 (BEI NR-56,461 Omicron variant), and hCoV-19/USA/MD-HP05285/2021 (BEI NR-55,671 Delta variant) was obtained from BEI Resources (Gralinski and Menachery 2020). All isolates were passaged up to four passages on Vero E6 cells (ATCC CRL-1586) and all stocks were diluted to 10^5.5^ TCDI/50/mL working solutions.

### Cell culture

Cell lines utilized included fetal deer testicle (FDTe) and Vero E6 cells (ATCC CRL-1586). All cell lines were maintained and cultured as previously described (Silveira et al. 2019). FDTe cells were harvested and derived at the USDA-ARS-National Animal Disease Center, Ames, IA from deer fetuses utilizing the previously reported methods described for harvesting and maintenance of bovine fetal testicle cells (Weber et al. 2017). Cells were seeded to achieve approximately 80% confluency after 24 h and then each cell line was inoculated with 10^5^ TCDI/50/mL with each variant to include single infection with Alpha, Delta, or Omicron variant and dual infection with Alpha/Omicron or Delta/Omicron. Non-inoculated cell lines were utilized to evaluate non-specific binding of the target probes. 24 h post infection, cells were harvested from each respective flask and plated onto 96-well round bottom plate.

### Flow cytometry analysis

**Fig. 1:**
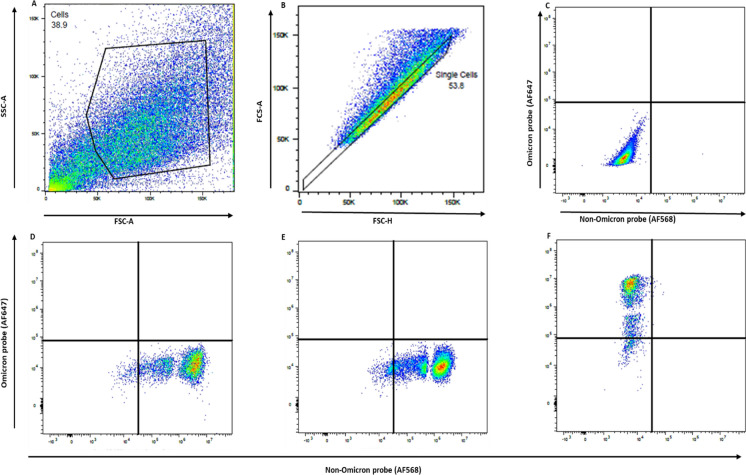
Evaluation of virus positive cells following 24-h inoculation using the PrimeFlow assay. Representative dot plots using Vero cell line showing **A** forward scatter area (FCS-A) vs. side scatter area (SSC-A) profile and **B** singlet discrimination by FSC-A vs. FSC-Height (H). Singlet events were then interrogated for detection of Omicron (AlexaFluor647 (AF647)) or non-Omicron (AlexaFluor568 (AF568)) positive and negative cells based on fluorescent signal from variant-specific probes. Representative dot plots for **C** control, non-infected Vero cells, **D** Vero cells 24 h post-inoculation with Alpha variant, **E** Vero cells 24 h post-inoculation with Delta variant, and **F** Vero cells 24 h post-inoculation with Omicron variant

Infection status was then assessed via PrimeFlow RNA assay, according to manufacturer’s instructions. Proprietary specific oligonucleotide (RNA) probes targeting the Spike protein of the SARS-CoV-2 variants were designed by Thermo Fisher Scientific. Two different target probes were designed to detect the Omicron variant (6008044-210 V) in the type 1 channel (fluorochrome AF647) or non-Omicron variants (6008045-210 V; Alpha and Delta variants) in the type 10 channel (fluorochrome AF568). Flow cytometric analysis was performed using a BD FACSymphony™ A5 flow cytometer (BD Biosciences). Compensation beads from the PrimeFlow kit as well as CompBeads (BD BioSciences, San Diego, CA) were used to set up compensation for each fluorochrome. While positive signals were evident, single stain controls and fluorescence-minus-one controls were evaluated to optimize acquisition gates and compensation for each fluorochrome/channel. Alexa Fluor (AF) 647 was excited using a red laser (637 nm). AF568 was excited using a yellow/green laser (561 nm). Cells were visualized in forward and side light scatter and electronic gates were placed on the scatter region that contained live cells. Doublet discrimination was then used to analyze single cells. At least 100,000 events were collected for each sample for data analysis. Data was collected and analyzed using FlowJo software as previously described (Silveira et al. 2019). Representative dot plots using Vero cell line showing FCS vs. SSC profile, gating strategy for singlet discrimination, and detection of Omicron or Non-Omicron positive and negative cells are illustrated in Fig. 1.

## Results

Positive signal was only observed when the respective virus and probe were both present, demonstrating the specificity for the target probes utilized in this study (Fig. 1). Non-infected cell cultures were used to test each probe individually and assess non-specific binding, and no positive signal was detected when non-infected cells were hybridized with each probe. Positive cells were only observed in both Vero E6 and fetal deer testicle (FDTe) cell lines, and frequencies of infected cells are reported in Table 1.

The greatest frequency of positive Vero cells in single infection treatments was observed for the Alpha variant (96%) followed by the Delta (95%) and Omicron (88%) variants (Table 1). Interestingly, during dual infection with Alpha/Omicron, no Vero E6 cells were positive for the Omicron variant, while 93% of the cells were positive for the Alpha variant as compared to 96% positive for single infection (Table 1). In dual infection with Delta/Omicron, 2% of the cells were positive for the Omicron variant and only 80% of the cells were positive for the Delta variant as compared to 95% positive for the Delta variant during single infection (Table 1). While a very small frequency of FDTe cells were positive, a similar trend was observed with Alpha variant having the highest frequency (3%), Delta (2%), and Omicron the lowest frequency (1%; Table 1). Only 1% of the FDTe cells were positive for the Alpha variant in the dual Alpha/Omicron infection and no positive signal for the Omicron. Lastly, only 1% of the FDTe cells were positive for the Delta and Omicron variant in the dual Delta/Omicron infection (Table 1).

**Table 1:** Frequency of cell lines positive for each respective viral probe

Cell line name	Virus strain	Sum of frequency of omicron probe+	Sum of frequency of non-omicron probe+
Fetal deer testicle	Alpha	0	3
	Alpha/Omicron	0	1
	Delta	0	2
	Delta/Omicron	1	1
	Omicron	1	0
Vero E6	Alpha	0	96
	Alpha/Omicron	0	93
	Delta	0	95
	Delta/Omicron	2	80
	Omicron	88	0

## Discussion

The goal of this work was to evaluate the PrimeFlow assay as a method to detect and characterize the frequency of SARS-CoV-2 variants at the single cell level as previously described for other viruses (Silveira et al. 2019). The PrimeFlow assay provided the ability to assess the positivity at the single cell level as determined by frequency of virus positive cells, in addition to dual positive cells suggestive of co-infection. Based on the frequency of virus positive cells, this data is also suggestive that the Alpha and Delta variants are more efficient at attachment and/or internalization compared to the Omicron variant, which to date is significant given the lack of models, methods, and data to understand these dynamics. Therefore, the leveraging in vitro methods, such as the one presented here, with other susceptible cell lines to SARS-CoV-2 or cell lines containing the human ACE2 receptor could yield more information about co-infection dynamics. The Caco-2 cell line has been reported to have little to no cytopathic effect after SARS-CoV-2 infection (Wurtz et al. 2021), which may provide the opportunity to further evaluate competition among variants over time without diminishing cell integrity over time. The Vero E6 and fetal deer testicle cell lines were chosen as Vero E6 is the prototypical cell line used for SARS-CoV-2 propagation and infection is observed within 24 h, whereas the fetal deer cell lines have also been described to be susceptible (Martins et al. 2022; Palmer et al. 2021), but detection of infection is prolonged compared to Vero E6, providing an opportunity to highlight the sensitivity of the PrimeFlow assay detection levels. The cytopathic effect of SARS-CoV-2 in cell lines and loss of cell integrity overtime is a limitation to using this assay to assess by flow cytometry as the duration of time post-infection is limited. Another opportunity to utilize this method would be to evaluate immune pressure using sera from previous exposure or vaccination to evaluate the potential of emerging variants to out compete prototypical strains. Additionally, while both Vero and FDTe cells were positive, it should be noted that SARS-CoV-2 isolates were propagated on Vero cells and therefore may explain the increased frequency observed in the Vero cells. Adaption of each variant to each respective cell line may be necessary prior to evaluation of infection dynamics associated with susceptible cell lines. Lastly, detection of variants is limited and is the rational for why only two target probes were used in the PrimeFlow assay (Omicron and Non-Omicron positive). The lack of genetic differences between the Alpha and Delta SARS-CoV-2 strains compared to the Omicron strain only provided the opportunity to specifically target the Omicron variant and the Alpha and Delta could not be distinguished and a Non-Omicron target probe was used for both of these variants. It should be noted that the Omicron has a greater number of mutations in the Spike protein (Rana et al. 2022; Rashid and Salih 2022) which was used for development of the target probes and is necessary for designing variant specific probes for differentiation and ensure no cross-reactivity. Data from this study shed light on initial infection dynamics in the absence of any immune pressure or adaptation of the virus on each respective cell line. Collectively, the data presented here are the first report utilizing the PrimeFlow assay as a method to characterize initial infection dynamics at the single cell level for SARS-CoV-2 variants.

## Data Availability

All data used in this study are available in the manuscript or any raw data will be made available upon request via email to the corresponding author.
